# The buried knot technique for all inside graft link preparation leads to superior biomechanical graft link stability

**DOI:** 10.1038/s41598-018-38150-w

**Published:** 2019-02-06

**Authors:** Thomas M. Tiefenboeck, Lena Hirtler, Markus Winnisch, Harald Binder, Thomas Koch, Micha Komjati, Marcus Hofbauer, Roman C. Ostermann

**Affiliations:** 1 Department of Orthopedics and Trauma Surgery, Division of Trauma Surgery, Medical University of Vienna, Vienna, Austria; 2 Department of Anatomy and Cell Biology, Medical University of Vienna, Vienna, Austria; 3 Institute of Materials Science and – Technology, Technical University of Vienna, Vienna, Austria; 4 Department of Orthopaedics, Sacred Heart Hospital of Jesus, Vienna, Austria

## Abstract

The aim of this study was to measure and compare the biomechanical properties of two different graft link preparation techniques for anterior cruciate ligament reconstruction. We hypothesised that there would be differences in elongation, load at failure and failure mode due to the different graft link preparation techniques. Thirty fresh-frozen anatomical specimen knees were used. Both tendons (semitendinosus and gracilis) were harvested and randomly assigned to two groups. Graft links prepared with a continuous loop technique were allocated to group 1, whereas those prepared with a buried-knot technique were allocated to group 2. The mechanical properties of both techniques were measured. A mean load to failure of 731 N and an overall graft elongation of 6 mm was found in the continuous loop group. In the buried-knot group, a higher load to failure (848 N) and a lower mean overall elongation (5 mm) was found. The buried-knot technique showed better results with significantly higher load to failure and significantly less elongation compared to the continuous loop technique. It is essential in clinical practice to choose the most accurate technique for graft link preparation to ensure graft stability, especially in the early phase of recovery.

## Introduction

Anterior cruciate ligament – reconstruction (ACL-R), utilizing autologous hamstring tendons, has become one of the most frequently performed orthopaedic procedures^1^. It requires special graft preparation techniques from the operating surgeon. The graft source, graft length and fixation method need to be considered for each patient^2^. Especially for the All-Inside ACL-R technique^1,2^, several graft preparation techniques have been described using different suture techniques^2–5^.

Depending upon the preferred reconstruction technique special graft preparation is necessary.

According to surgeon experience and clinic standards different techniques are used, all with the same goal of creating the “ideal” graft link.

The suture technique for graft link preparation is of great importance since it binds the graft together, is required for passing the graft through the tunnels and needs to withstand the tension forces during implantation as well as in the first months of implant ingrowth. Forces affecting the graft, either during graft implantation or in the early postoperative phase, until complete ingrowth into the bony tunnel, may cause clinical failure, which is of significant concern^6–9^. During daily activities, the anterior cruciate ligament (ACL) is exposed to forces of approximately 156 Newton (N) to 170 N, this can increase to 448 N by going downstairs^10,11^ and up to 700 N during stumbling, which can cause graft failure in an early stage after reconstruction. These forces need to be considered when using different graft link preparation techniques.

Several factors have been shown to potentially affect graft elongation, e.g. tissue quality, stitch technique, suture strength^12^, and the number of used suture throws^6–9^. An increase in the number of sutures across the tendon graft may provide more contact area between the thread surface and the tendon to ensure security of the construct. However, using more sutures results in a higher amount of material near the joint, which could irritate the joint or increase the risk of infection^13,14^. Successful ACL reconstruction with a tendon graft requires solid healing of the tendon graft in the bony socket, which itself requires adequate graft fixation initially as well as tight press fit between graft and bone socket.

To enhance graft-tunnel healing, tissue-engineering approaches, including the use of growth factors, mesenchymal stem cells, and periosteum graft augmentation, have been used showing promising results in terms of enhancement of bone-graft healing rate^15^.

However, literature lacks comparisons of different suture techniques for the All-Inside ACL-R technique.

The purpose of this study was to measure and compare the biomechanical properties (elongation, load to failure and mode to failure) of two different ACL graft link preparation techniques. We hypothesised that there would be differences in elongation, load at failure and failure mode due to the different graft link preparation techniques. The graft link preparation using more sutures through the tendon construct will result in a more stable graft with regards to tendon strength and elongation.

## Methods

Thirty fresh-frozen anatomical specimen knees (13 woman, 17 men) were obtained from the Department of Anatomy and Cell Biology, Medical University of Vienna. The donors had a mean age at death of 68 years (range, 50 to 72 years; STD, 5 years). The biomechanical testing was carried out at the Institute of material science and technology, Technical University of Vienna, under the guidance of a specialist in material science.

### Graft Harvesting

All included knee specimens were stored until harvesting at 4 °C in a designated refrigerator at the Department of Anatomy and Cell Biology, Medical University of Vienna. Through a medial incision semitendinosus- and gracilis tendons were harvested from all included knees to ensure protection of the tendons. All tendons were measured and included if they provided a minimum length of 270 mm and reached final diameter of minimum 8 mm after quadrupling. To measure tendon length a standard ruler was used. To measure tendon diameter the tendon-measuring block (Arthrex, Naples, FL) was utilized. Finally, a total of 24 grafts (10 women, 14 men), with 12 in each group of equal lengths were randomly assigned. Prior to inclusion, macroscopically degenerative or pathological changes were ruled out.

### Graft Conservation

After harvesting, included tendons were fresh frozen (−20 °C) until final testing. Before testing they got moved from the freezer to the refrigerator to ensure slow thawing. Finally they were kept at room temperature (21 °C). The whole procedure takes 36 hours before testing. Slow thawing ensures that there is no dehydration. Before and during the preparation all tendons were humidified with 0.9% sodium chloride to counter dehydration.

### Preparation of Graft Link

**Figure 1:**
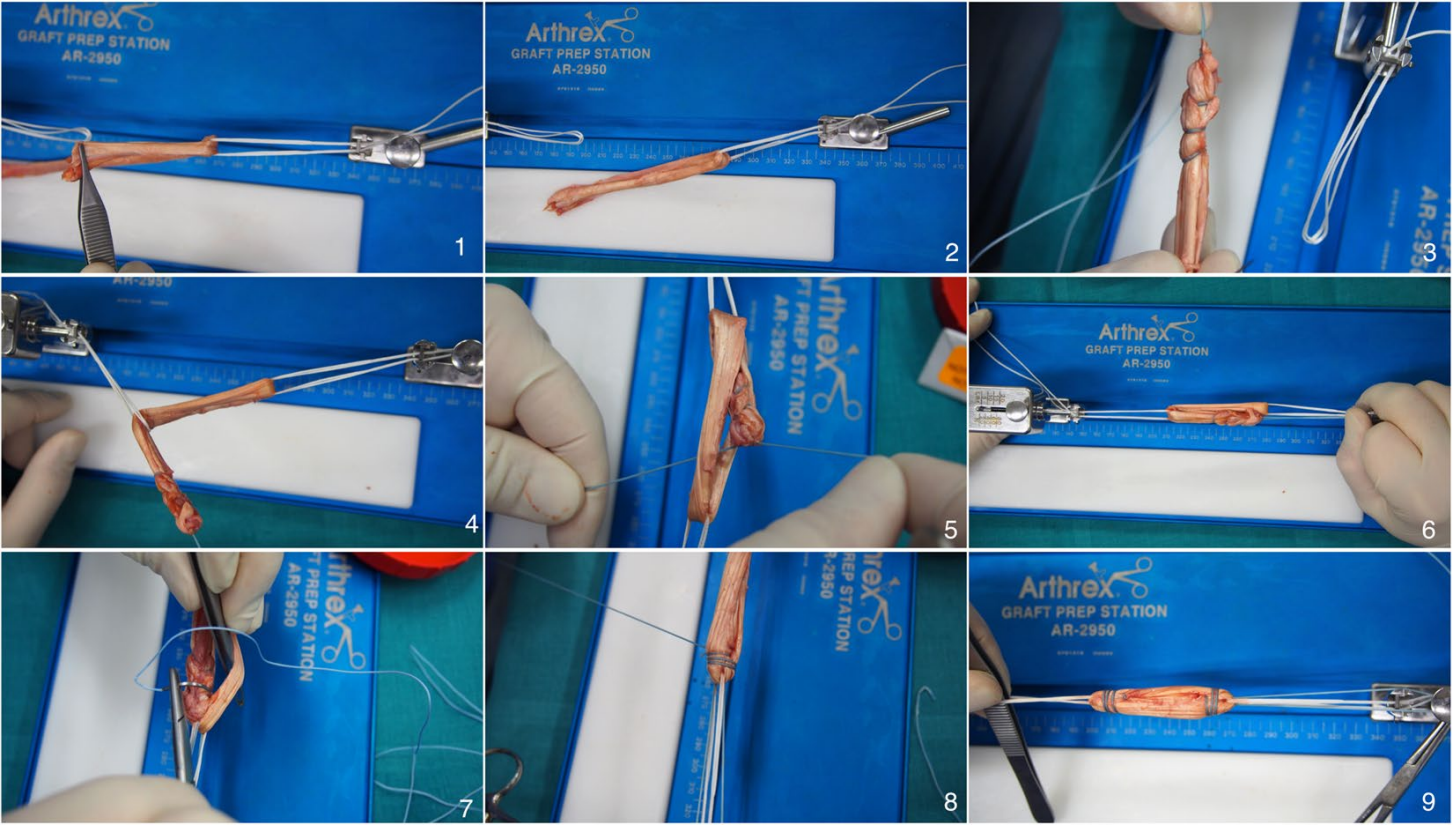
Presents the all-Inside graft link preparation with a semitendinosus graft (buried-knot technique) as shown by Lubowitz in 2012. (Logo used with permission of Arthrex) (1) The free ends of the graft are passed to the same side of the loop. (2) Both ends are than brought together (3) to get whipstiched together with No 2. FiberLoop. (4) Then the whipstiched part is passed through the other TightRope (5) and brought between the tendon. (6) The two ends of the FiberLoop were tensioned and fixed on the graft preparation station. (7), (8) Next, the graft is baseball stitched on both ends with a No. 2 FiberWire creating the final graft link. Each stitch is passed through each of the 4 strands of graft and the suture free limb is wrapped once around the collagen bundles, creating a self-reinforcing suture noose. (9) Represents the final graft link.

**Figure 2:**
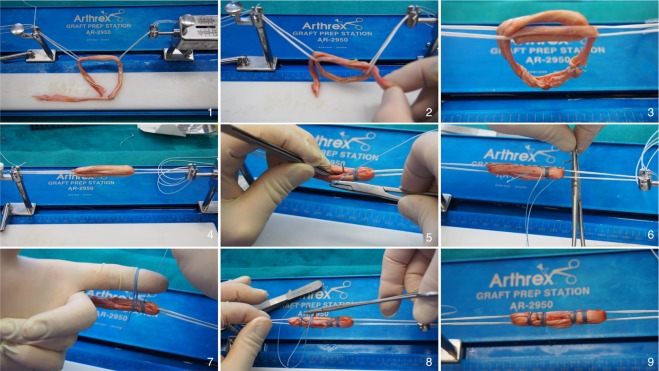
Showing the continuous loop technique using a quadrupled semitendinosus tendon Graft for ACL graft link preparation. (Logo used with permission of Arthrex). (1) First the ACL TigthRopes are loaded onto the graft preparation station. The end of the semitendinosus graft is passed through the first ACL ThightRope and than through the second, creating a short length and a long length. (2) The longer end is than brought back through both ACL TightRopes to create a quadrupled graft. (3) The two free ends are sutured together using a FiberWire with one to two running baseball stiches. (4) Then the graft is slightly tensioned and the knot is put to the inner loop. (5), (6), (7), (8) Than the graft is sutured, in a standard way, with a number 2 FiberWire. Starting on the inside passing through two limbs then the suture is passed three to four times around the graft. The needle is pooled back in the middle of the graft through the other two limbs and than tight in a standard way. (9) This procedure is repeated four times to get the final graft link.

Two authors (TT and MW) solely performed all graft link preparations. Each graft link preparation was timed. The graft preparation and tension device (Arthrex, Naples, FL) was utilized for preparation of all tendons. Two different preparation techniques were performed in this anatomic specimen study: Group 1: the buried-knot technique presented by Lubowitz J. in 2012^2^ (Fig. 1); and Group 2: tendon preparation using the continuous loop technique (Fig. 2). Twelve tendon pairs (semitendinosus and gracilis) were each prepared according to one of the two techniques, matching the inclusion criteria described above.

**Figure 3:**
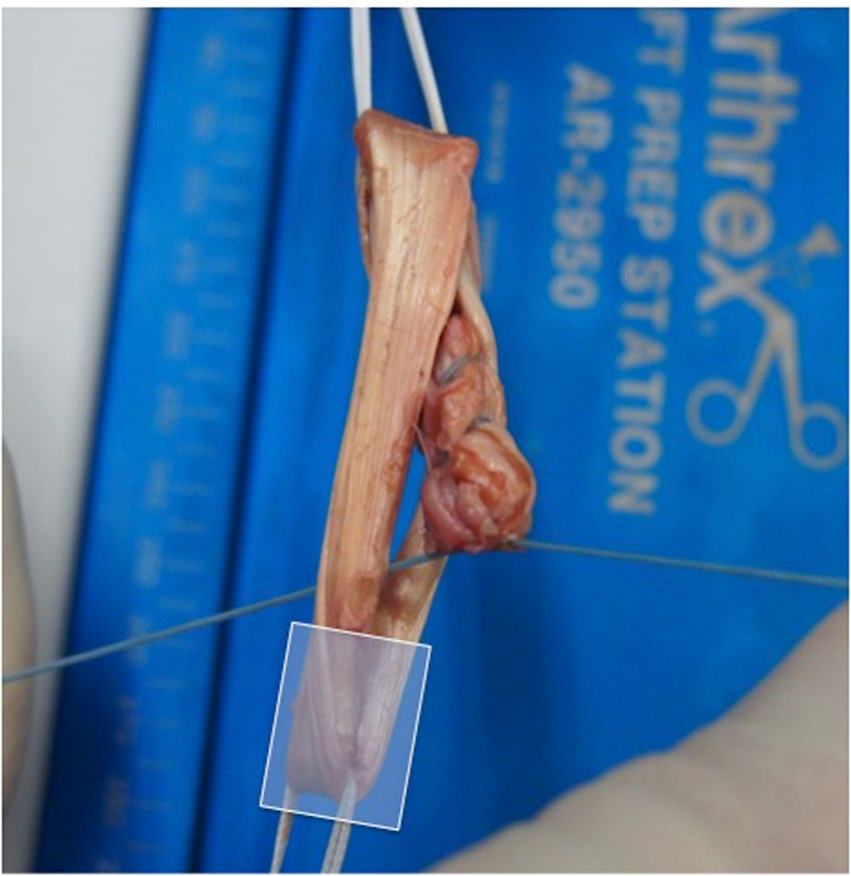
Presenting the definition of the femoral part of the buried-knot technique. (Logo used with permission of Arthrex). In every graft link, femoral part was defined, as part where only one handle of the tendon was put trough the TightRope as marked in the figure.

**Figure 4:**
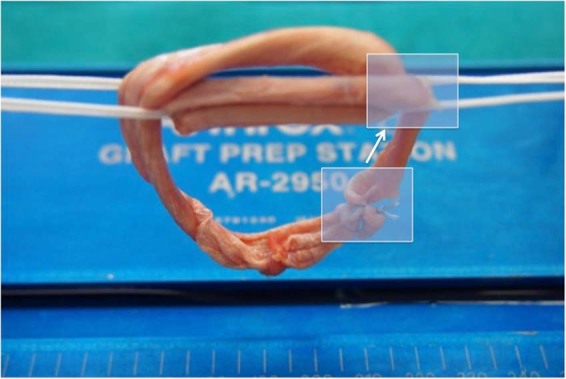
Presenting the definition of the femoral part of the continuous loop technique. (Logo used with permission of Arthrex). Femoral part was defined, as part where the primary fixation was set as marked in the figure.

In each graft link a femoral and tibial diameter was defined to be able to name the area of graft failure. In the buried-knot technique the femoral part was defined as the part where only one handle of the tendon was brought through the TightRope (Fig. 3). In the continuous loop technique the part where the primary fixation was put between the tendon handles and sutured later was defined as the femoral part (Fig. 4).

In both groups a No. 2 FiberWire (Arthrex, Naples, FL) suture was used. An additional No. 2 FiberLoop (Arthrex, Naples, FL) suture was used in the buried-knot technique^2^. All hamstring grafts were fixed with the TightRope (Arthrex, Naples, FL) system at the material testing machine, between two metal plates through a hole representing the button flipped over the cortex.

During the preparation all tendons were humidified with 0.9% sodium chloride as a routine procedure at the Department of Orthopedics and Trauma Surgery, Medical University of Vienna. A total of 24 tendons, after ruling out macroscopically degenerative or pathological changes, and final grafts of equal length ±10 mm and a diameter ±1.5 mm, were prepared. The final graft link (tibial diameter, femoral diameter) was measured with a sliding calliper. For graft link measurement a standard ruler was used.

### Measurements

**Figure 5:**
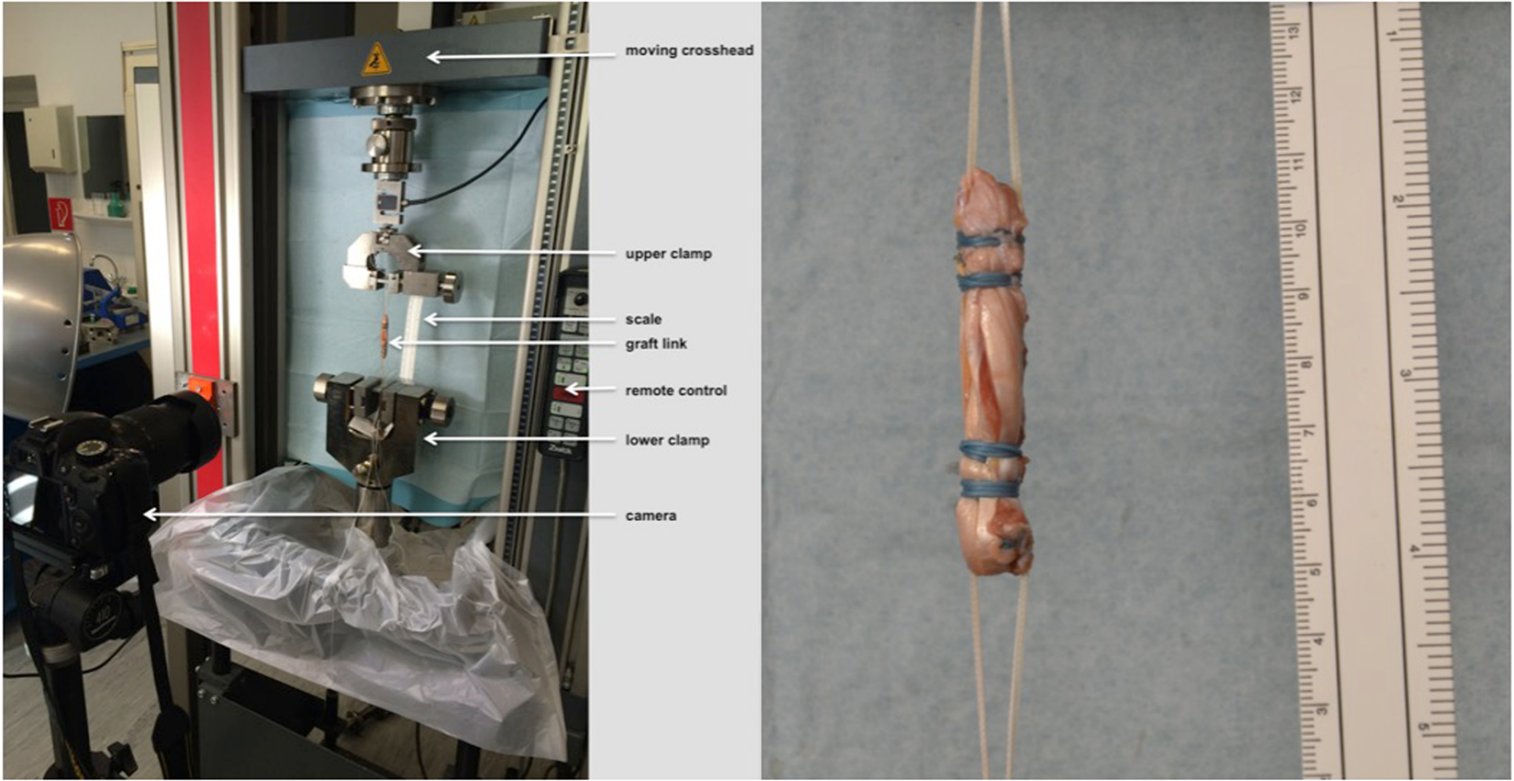
Method used to test the graft link preparation technique. The graft link is attached to the materials testing system (Zwick Z050, Zwick Roell, Germany). (left side) General view, (right side) close-up view of the graft link.

A digital single lens reflex camera, type Nikon D500, was used to take photographs. All grafts got pre-tensioned at the graft preparation system by Arthrex Naples, FL, before loading at the testing machine. Both ends of each single graft were secured with the ACL TightRope (Arthrex Naples, FL) system, fixed through a metal plate, which was attached at the Zwick Z050, universal material testing machine (Zwick Roell, Germany). The Zwick Z050 was loaded with a 1 N load cell and a video extensometer (Fig. 5.) The initial distance between the two TightRopes was calculated to be 60 mm ± 10 mm. The elongation of the tendons was measured based on the motion of the crosshead of the testing machine, additionally the elongation was measured with the ruler.

After initial loading of the graft link (1 N) onto the Zwick Z050 a photograph was taken to measure the primary length of the graft link. 50 N at a rate of 200 mm/min for 10 cycles was applied to the graft link to mimic the tension of sutures tied over a cortical bone bridge. The strength of the graft link was tested by applying 50 to 250 N cyclic loading for a total of 500 runs. This testing procedure is a standard protocol utilized in previous studies^6,16,17^.

### Statistical Analysis

**Figure 6:**
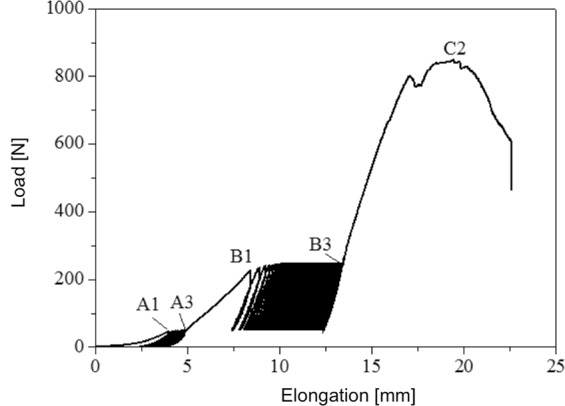
Presents a sample load-elongation curve of a graft link preparated with the buried-knot technique. The pretensioning (A1–A3), total elongation (B1–B3) and load to failure (C2) can be seen.

The statistical analysis was performed using Excel 2014 (Microsoft, Redmont, WA) and SPSS (Version 23.0, SPSS Inc., Chicago, IL, USA) software packages. Regions of interest (ROI) were defined before final testing. (1) A1 to A3 - area of pretensioning, (2) B1 to B3 - total elongation during the cycling load, (3) C2 - load to failure and (4) mode to failure. (Fig. 6. present ROIs in detail).

For the whole cohort a descriptive statistic was performed (means, medians and standard deviations). For comparison of two metric variables, a student’s t-test was used.

Normal distribution of metric variables was verified by application of the Shaphiro-Wilk test. If the Shaphiro-Wilk test was rejected, the Mann-Whitney U test was used. Tests were used to compare (1) suture and tendon percentage elongation with pre-tensioning, (2) cyclic loading, (3) load to failure and (4) stiffness. A significance level was set at p ≤ 0.05. As this study was a pilot study no power analysis was performed.

### Ethical approval and informed consent

Prior to study begin the corresponding ethic review board, Medical University of Vienna, approved the study (EK 1214/2015). All patients signed informed consent that they donate their body to science after death. All used methods were carried out in accordance with the relevant guidelines and regulations of the institutions involved.

The suture material, TightRopes and the working station were provided by Arthrex for free (Naples, FL). Parts of the presented data (group of continuous loop technique) have been published previously in a paper from the first author. (Tiefenboeck, T. M. et al. A bigger suture diameter for anterior cruciate ligament all-inside graft link preparation leads to better graft stability: An anatomical specimen study. The Knee 25, 427–433, (2018) 10.1016/j.knee.2018.03.010).

## Results

The total graft preparation time (mean 7 minutes) was comparable in both groups, not showing significant differences (p = 0.873). After applying 1 N for measuring initial tensioning, no differences were found between tested techniques (p = 0.7298). Detailed overview of data for the continuous loop technique and the buried-knot technique is presented in Table 1.

**Table 1:** Descriptive statistics for the biomechanical properties of the continuous loop and the buried-knot technique for ACL graft link tendon preparation.

	Continuous Loop (n = 12)	Buried-knot Technique (n = 12)	p-value
Mean tibial diameter (mm)	9.7; median; 9.8, range; 8.5–10.5; SD ± 0.59	9.1; median; 9; range; 8–10; SD ± 0.64	0.037
Mean femoral diameter (mm)	9.6; median; 9.8, range; 8.5–10.5; SD ± 0.67	9.3; median; 9; range; 8.5–10.5; SD ± 0.66	n.s.
Mean load to failure (N)	731; median; 709, range; 619–936; SD ± 95	848; median; 844.1 range; 731.4–955.5; SD ± 56	0.002
Mean overall elongation (mm)	6; median; 5; range; 4–10; SD ± 2	5; median 5 range; 4–6; SD ± 0.5	0.045

n.s. = non significant; mm = millimetre; N = Newton, n – number, SD – standard deviation.

**Figure 7:**
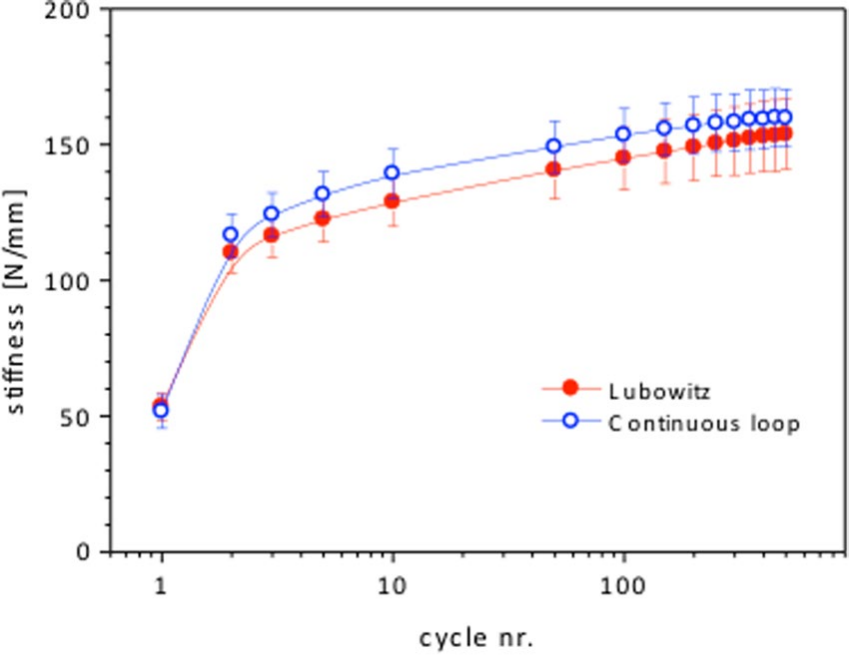
Shows the stiffness of the two different techniques in regard to the cycles.

The measured load to failures showed significant differences, revealing the buried-knot technique to be stronger (p = 0.002). There were also differences found in the overall elongation with the buried-knot technique showing a significantly lower elongation rate (p = 0.045). However, the tibial diameter of the grafts was significantly lower (p = 0.037) in the buried-knot technique group; differences regarding the femoral diameter were not found. With regard to measured stiffness there was no significant difference found (p = 0.4631), however the continuous loop technique presented with better values regarding stiffness (Fig. 7).

**Figure 8:**
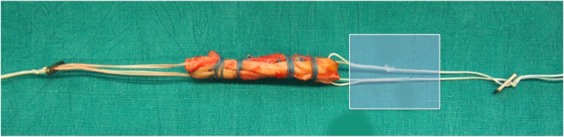
Shows the part of TightRope crossing; were the failure occurred most often.

The most common mode of failure (75%) in the buried-knot technique was rupture of the TightRope “crossing” after an average load of 864 N ± 40 N (median, 845 N; range, 822 to 955 N). The TightRope “crossing” was defined as the part of the TightRope where tightening is possible (Fig. 8). In two grafts (17%), the TightRope cut through the tendon (894 N and 774 N) and in one graft (8%), the running baseball stiches cut through the tendon with a load to failure of 731 N.

The most common mode of failure in the continuous loop technique was loosening of the baseball stich (Fig. 2.3), which was only used as primary fixation to be able to load the tendon on the tendon preparing facility for easy preparation of the graft. In 51% (N = 6) this specific primary fixation failed, after an average load of 745 N ± 79 N (median, 746 N; range, 619 to 849 N). The FiberWire cut through the tendon in 42% (N = 5) after an average load of 673 N ± 45 N (median, 643, range, 634 to 746 N) and in one graft the TightRope ruptured at the “crossing” after 935 N.

## Discussion

In summary, we found a significant higher load to failure in grafts prepared according to the buried-knot technique compared to the continuous loop technique presented. Furthermore, there was a significant difference in the rate of elongation of the tendons detected, showing the tendons prepared by the buried-knot technique to elongate significantly less (mean 4.7 mm to 5.9 mm; p = 0.045) compared to the continuous loop technique. This might be due to the fact that all tendons in the buried-knot technique were stitched three to four times more often, with baseball stiches, compared to the continuous loop technique. In the continuous loop technique, tendons were only stitched once and the graft was then surrounded by FiberWire (four times, twice on each side). This could also be the reason for easier cutting through of the tendon compared to the buried-knot technique.

Interestingly, as we found the buried-knot technique to present with a significant higher load to failure, in this technique only one handle of the tendon is loaded through a TightRope (defined as the femoral site, Fig. 1.2), the other handles are placed between (Fig. 1.5) and then fixed with baseball stiches (Fig. 1.7), revealing the tendon to be very strong, suggesting that graft failure might be caused by other factors. Boniello *et al*.^18^ showed that by using greater graft diameters a higher strength could be reached, leading to the conclusion that even a tendon with a diameter of 6 mm produces an average load to failure of 2358.8 N. This is in line with our results where tendon failure was rarely seen.

Regarding the current literature, several studies investigated different knot techniques^16,17,19–21^, or number of used knots^6^, however, this is the first study investigating biomechanical differences of two ACL graft link preparation techniques in human specimens. By using the ACL all-inside ACL-R technique a detailed knowledge of the preparation technique is necessary and therefore should always be performed by an experienced surgeon or under direct supervision.

Before graft link preparation, suturing and tensioning, graft specifications need to be followed in both techniques. Graft length should not be greater than 270 mm in length to avoid an overlengthening of the graft after pretensioning. Lubowitz recommended a graft diameter with a maximum of 8.5 mm^2^. However, from our clinical experience, graft diameter should be at least 8 mm to avoid early graft failure.

In summary, there are many factors presented in literature influencing final graft link stability and the risk of revision surgery in ACL-R^22,23^. Ruling out the most crucial ones is very hard and further work is needed. In the MARS cohort, technical faults were presented in 24% of the patients leading to ACL revision^23^. Reasons for this are diverse also including inadequate graft link preparation.

There are limitations to any biomechanical study. Firstly, although all tendons were checked macroscopically for any damage or failure, microscopical differences in tendon quality could not be ruled out and might influence outcome. Furthermore, only fresh frozen anatomic specimens were used for this study. Consequently, it would be advisable to repeat this study with fresh human anatomical specimen semitendinosus and gracilis tendons. Despite this limitation, there was unanimity amongst the authors with regards to the included tendons, which were finally used for the experiment. All tendons were randomly assigned to one of the two groups. The grafts were not implanted into bone, as done during actual surgery, so it is not possible to know how these preparation methods perform after fixation in bone, which is a critical issue.

Secondly, this study focused on tendon elongation and load to failure without considering the physiological healing of the tendon and the tendon-bone construct, which could influence to the success of tendon graft reconstruction surgery. This needs to be considered when interpreting the results.

Thirdly, for graft link testing cyclic loading was used only in an axial direction, not considering any rotational movement, which would be normal in physiological conditions. Reconstruction of the ACL using tendon grafts often leads to compacting and shear forces between the tendon and the bone, which is sophisticated to stimulate in an *ex vivo* biomechanical setting. Nevertheless, this is the first study using human graft link constructs in an *ex vivo* setting examining the performance of graft link constructs by comparing two different preparation techniques.

## Conclusions

The buried-knot technique yielded significant improvement of the load to failure and significant reduction of elongation compared to the continuous loop technique. It is essential in clinical practice to choose the most accurate graft link preparation technique to ensure the highest graft stability, especially in the early phase of recovery. However, further *in-vivo* studies are needed, including bony ingrowth of the tendon, physiotherapy protocols, e.g. influencing early graft link stability.

## Data Availability

The data that support the findings of this study are available from the corresponding author upon reasonable request.
